# A machine learning model for assessing fetal health during pregnancy

**DOI:** 10.3389/fbioe.2025.1691064

**Published:** 2025-12-17

**Authors:** Arshad Kalathil Ashik, Robert Gutierrez, Fidha Ashraf, Tricia Adjei, Sohini Patel, Isabella Abati, Zhenhua Yu, Saksham Dhawan, Jia Li, Khondaker A. Mamun, Thomas Reddyhoff, Daniele Dini, Christoph Lees, Ravi Vaidyanathan

**Affiliations:** 1 Department of Mechanical Engineering, Imperial College London, London, United Kingdom; 2 Department of Psychiatry, Lancashire Care NHS Foundation Trust, Preston, United Kingdom; 3 Institute of Reproductive Developmental Biology, Department of Metabolism Digestion and Reproduction, Hammersmith Campus, Imperial College London, London, United Kingdom; 4 Department of Computing Science, University of Aberdeen, Aberdeen, United Kingdom; 5 Department of Computer Science and Engineering, United International University, Dhaka, Bangladesh

**Keywords:** acoustic sensing modalities, fetal movement, FM, piezoelectric, rusboost model, vibrational-acoustic sensor array, vibroacoustic sensor array

## Abstract

There is a global imperative to end stillbirths, particularly in low middle income countries (LMICs), which suffer from disproportionate incidence. Sudden changes in fetal movement (FM) patterns often precede a crisis, which, if flagged, can trigger life-saving intervention. Existing means of FM tracking, however, are based on outdated understanding which have remained unchanged for decades. The current standard for monitoring FM out-of-clinic remains maternal perception, which suffers from subjectivity and has little impact in reducing stillbirth or poor perinatal outcomes. Ultrasound can trace FM and trigger intervention but is administered sporadically over the course of pregnancy and demands clinician expertise and resources; frequent use is not feasible, particularly in LMICs. Wearable FM monitors have been proposed for FM empirical movement monitoring, however, clinical impact remains negligible due to homogeneous sensing modalities and lack of clinical validation. Herein, a multimodal vibrational-acoustic sensor array consisting of piezoelectric and acoustic sensing modalities is validated for use in a wearable FM. 25 pregnant participants were recruited to record vibrational-acoustic data from the array in parallel with ultrasound scanning. Categorised fetal movements were recorded by a clinician, and several machine learning models were investigated to validate the sensors to track FM. An ensemble RUSBoost model combined with concatenated sensor data inputs was implemented, yielding FM prediction with precision and recall of 0.44 and 0.61, demonstrating the feasibility of the vibroacoustic sensor array to monitor FM. Inexpensive off-the-shelf sensors comprising the array provide a basis for the development of a fully wearable FM monitor that can be used in LMICs.

## 1 Introduction

The sustainable development goals established by the United Nations in 2015, aim to globally end preventable stillbirths and neonatal deaths by 2030 (World Health Organization, 2015). While stillbirth rates have been steadily falling since at least 1990 (World Health Organization, 2015), an estimated two million stillbirths in 2019 (Hug et al., 2021) suggests we are still a long way from this target. There are large regional variations in stillbirth rates, with 22.8 stillbirths out of 1,000 total births in central and west Africa, where as a number as low as 2.9 was estimated for western Europe (Hug et al., 2021). Many countries in sub-Saharan Africa, East Asia, Central America and the Caribbean have not reduced their stillbirth rate since 2000 (Hug et al., 2021).

These studies recognise there is significant under-reporting in low -income countries therefore substantial under-estimation in the global stillbirth rates (Christou et al., 2017). Nevertheless, the quantifiable rates of global stillbirth highlight the inequality in access to healthcare (de Bernis et al., 2016) demonstrating the need for change in fetal health monitoring.

Maternal perception of fetal movement remains the most common way to monitor fetal health out-of-clinic before birth. A decrease in perceived fetal activity may be associated with poor fetal outcomes (Berbey et al., 2001). Previous studies have indicated that pregnant women are aware of fetal movements (Saastad et al., 2008). However, educational differences amongst populations make understandings of maternal perception hard to unify (Weller et al., 2023). More than a third of women do not recall receiving information on the importance of reporting decreased fetal activity (Bradford et al., 2019). Additionally, definitions of a normal number of perceived movements vary depending on which country you receive care, with some countries advising from 25 kicks per hour to three kicks per 24 h (Saastad et al., 2008). In the United Kingdom kick counts, charts and prescriptive amounts of movements are out of practice (Callaghan, 2018). Therefore, individualising care is paramount.

Some investigations (Bradford and Maude, 2018; Hijazi and East, 2009) have noted that women tend to feel an increasing strength in movements as gestation increases. There are also many factors which influence maternal perception of fetal movement, such as gestational age, overweight/obese, sedatives and other drugs (Bradford and Maude, 2018). Many movements are unperceived, therefore maternal perception of a decrease in fetal activity does not necessarily correlate to a reduction in fetal activity (Hijazi et al., 2010). While women can self-monitor fetal activity to some degree, this is a subjective measure of activity (Berbey et al., 2001). Therefore, a more rigorous method to quantify and monitor fetal activity would be advantageous.

Ultrasound imaging provides objective fetal movement monitoring. It is a non-invasive method and can detect many movements which are not detected by maternal perception. A study on fetal maternal perception (Hijazi et al., 2010) found that a mother could only detect 36% of all movements detected by ultrasound scans. However, there are disadvantages associated with ultrasound imaging. Ultrasound equipment is often large and expensive, meaning it is not affordable in many low middle income countries (LMICs) and rural areas, precisely areas which are most prone to preventable stillbirths (Harvey et al., 2014). Moreover, this equipment can only be undertaken by a trained practitioner. In a survey conducted to assess the barriers to using ultrasound in resource-limited settings (Shah et al., 2015), it was found that a lack of training was the main reason given by healthcare workers for not being able to use ultrasound equipment effectively. Equipment maintenance is another challenge for healthcare providers in rural areas, as trained technicians are often not available to do regular maintenance (Shah et al., 2015). Ultrasound scans require a patient to attend a hospital appointment. Which is not always practical in both high and LMICs. Additionally, ultrasound is used sporadically for fetal movement monitoring as it cannot be used for prolonged periods due to possible adverse effects. Many studies have explored the effects of ultrasound monitoring on fetal health (Shankar and Pagel, 2011; Rodgers, 2020; Torloni et al., 2009). While there is currently no clear correlation between fetal health and ultrasound monitoring, the possibility of adverse effects cannot be ruled out and a conservative approach should be taken when applying ultrasound monitoring.

The concept of wearable fetal movement monitors has been proposed in several studies as a means of empirically tracing fetal movements [surveyed recently in (Patel et al., 2025)]. In recent years several acoustic/accelerometer-based wearable home testing techniques has been proposed (Nishihara et al., 2008; Ghosh et al., 2024; Valentin et al., 1986; Ryo et al., 2012). Validation, however, remains a challenge as there is no means, other than maternal perception, to label an accurate fetal movement count out-of-clinic; home testing alone remains uncertain. A smaller proportion of studies have investigated the effectiveness of these sensors in detecting fetal activities compared to clinical ultrasound. A study conducted on two custom made capacitive accelerometers to record fetal movements and compare them against clinician perceived ultrasound reported a prevalence-adjusted bias-adjusted kappa (PABAK) value of 79% for gross movements and 36% for isolated activities (Nishihara et al., 2008). Another study investigated four piezoelectric crystals to detect fetal movements compared to movements sensed by the mother, achieving a sensitivity (recall) of 78%. However, the system produced a high number of false positives, resulting in a precision of 40% (Valentin et al., 1986). While the majority of fetal movement monitors have been executed with a single sensing mode, recent work (Ghosh et al., 2024) has proposed a heterogeneous sensing suite consisting of piezoelectric, acoustic, and accelerometer sensors. Home testing showed complementary features of sensor response led to greater detection accuracy, however testing was still only done in comparison to maternal sensing out-of-clinic. Despite this promise, these studies were conducted in a controlled environment and analysed and categorised fetal movements separately (gross and fine movements). However, relying only on gross activity accuracy does not provide complete assessment of fetal health. There are a range of physician classified fetal movements, including breathing, startle, and limb movements which can be relevant to the health of the fetus (Patel et al., 2025). While detecting this entire range may not be feasible for a wearable monitor, a broader set of movement classifications, even two classes, could significantly aid in fetal health monitoring.

This work aims to build a tuneable Machine Learning (ML) framework that can analyse both fine and gross fetal activities for a heterogeneous array with sensors of varying frequency response to capture a finer range of fetal movements. We implement the system on a heterogeneous array of vibrational-acoustic sensors and validate the movement prediction of the framework against clinician labelled ultrasound. Three piezoelectric and three custom packaged acoustic sensors were used to record data from expectant mothers. The performance of these sensors to trace fetal movement was compared against clinical ultrasound scanning. Testing involved a 30-min ultrasound scan session where this sensor suite was attached to the participant’s abdomen. Fetal movements were called out by the sonographer in real time. This was used as the gold standard comparator to identify movements on the recorded vibrational-acoustic signals. Finally, an ML model was developed to analyse and identify fetal movements from all six sensors for each of the 25 participants. The model architecture provides a basis for tuning sensor arrays for multimodal fetal movement monitors.

## 2 Experimental setup

25 pregnant participants were recruited in a tertiary maternity unit in London, in the United Kingdom. All participants chosen were above the age of 18 and between 24 and 38 weeks of gestation. To better reflect the spread of a true population, participants of mixed risk were recruited by ensuring that each met one or more criteria from a list of high-risk categories. These categories included a high body mass index (BMI) greater than 35, diabetes, hypertensive disorders of pregnancy, obstetric cholestasis, smoking at the time of discovering the pregnancy, advanced maternal age above 40 years, small for gestational age fetuses, and a history of previous fetal death after 28 weeks, defined by the World Health Organization as stillbirth. All details of participant health collected before screening is reported in the Appendix section.

**FIGURE 1:**
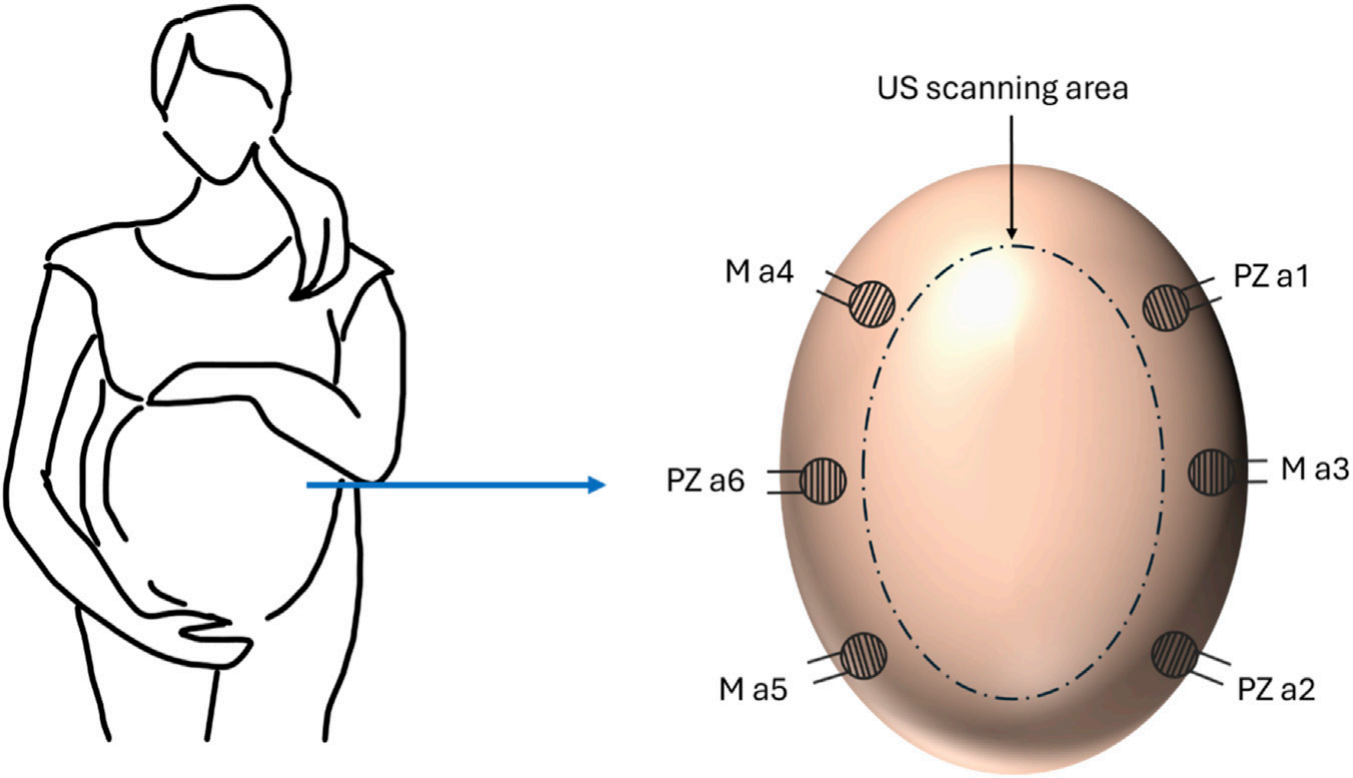
Sensor placement on the woman’s abdomen during ultrasound scanning (US). PZ refers to a piezoelectric sensor, M refers to an MMG sensor, and a(x) is for numbering.

Participants underwent ultrasound tests at 32–34 and 36–38 weeks of gestational age. However, due to various factors such as problems collecting data and patient availabilities, not all participants were able to do two scans. As a result, 38 scans of data were collected for analysis. Ethics has been approved and granted by the Health Regulation Authority (ref 23/PR/0859). Before each ultrasound scan, three piezoelectric sensors (PZ a1, a2, and a6) and three acoustic sensors (M a3, a4, and a5) manufactured in-house (Woodward et al., 2017) were attached to the pregnant woman’s abdomen as shown in Figure 1.

All six sensors used in this study are highly sensitive and can pick up small vibrations. During testing sessions, the ultrasound probe movement produced additional noise in these signals. To overcome this issue an inertia measurement unit (IMU) sensor was placed on the ultrasound scanning probe. The IMU recorded the ultrasound probe movements as it moved across the abdomen. Later the IMU signals were synced with the sensor signals to remove this noise. Additionally, to capture perceived fetal movements, a push button connected to the data logger was provided to each participant. While scanning, the participant was asked to press it whenever they perceived a movement.

Crucially, to train the ML algorithm to associate fetal movements with the attached sensor, ultrasound detected fetal movements were used as the gold standard of those movements detected in real time. This information was separately analysed to later identify the corresponding signal pattern. The four types of activities recorded were general movements, breathing, startle and limb movement. To have enough data for ML training all the ultrasound tests were done for a period of 30 min.

## 3 Data analysis

### 3.1 Data pre-processing

The raw signals obtained from the sensors contain significant noise and offset, which necessitates proper processing to extract meaningful information. The first pre-processing step was to remove the signal offsets by subtracting the signal mean value from each respective signal. This ensured that the inherent noise offset from the sensors was effectively cancelled out. It was also noted that the sensor signals have widely varying signal amplitudes. To make signals from different sensors more comparable, signal normalisation was required. This was achieved by dividing each signal by its root mean square (RMS) value.

Another source of noise was the disturbance introduced by the movement of the ultrasound probe during scanning. Sensors in close proximity to the probe detected higher levels of noise. To address this issue, the IMU data was analysed and a threshold was set to identify IMU movements. Then the sensor signal noise values corresponding to the IMU movements were set to zero, effectively cancelling them out.

To understand the sensor signal pattern corresponding to the fetal activity, the Ultrasound Detected Fetal Movement (UDFM) data was used. To synchronise the UDFM data with the sensor signals, the previously mentioned push button was trial pressed at the beginning of the scanning. Later when pre-processing the data, the time stamp from the push button was used to synchronise all signals.

**FIGURE 2:**
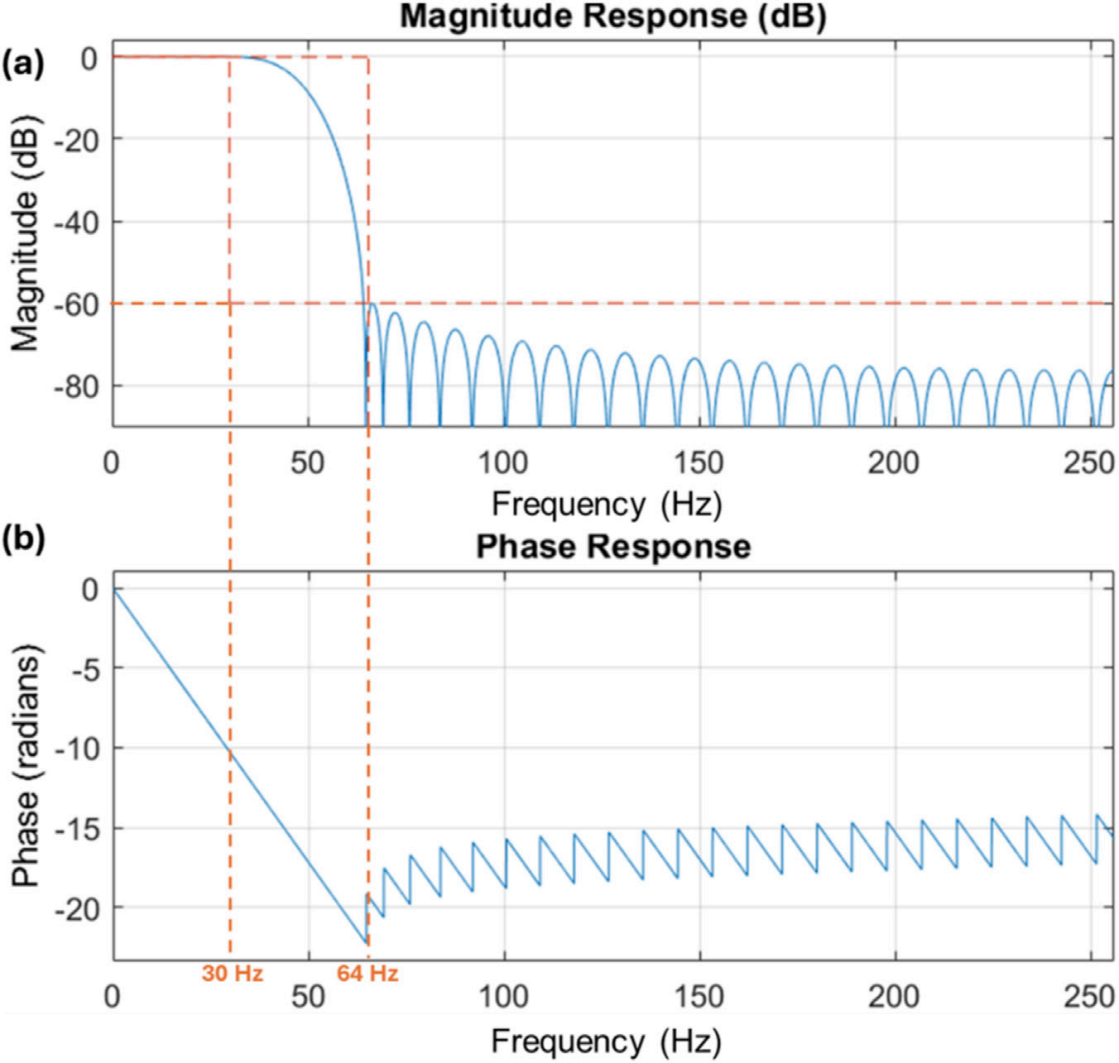
Bode plot for the low pass filter. **(a)** Filter Magnitude Response. **(b)** Filter Phase Response.

**FIGURE 3:**
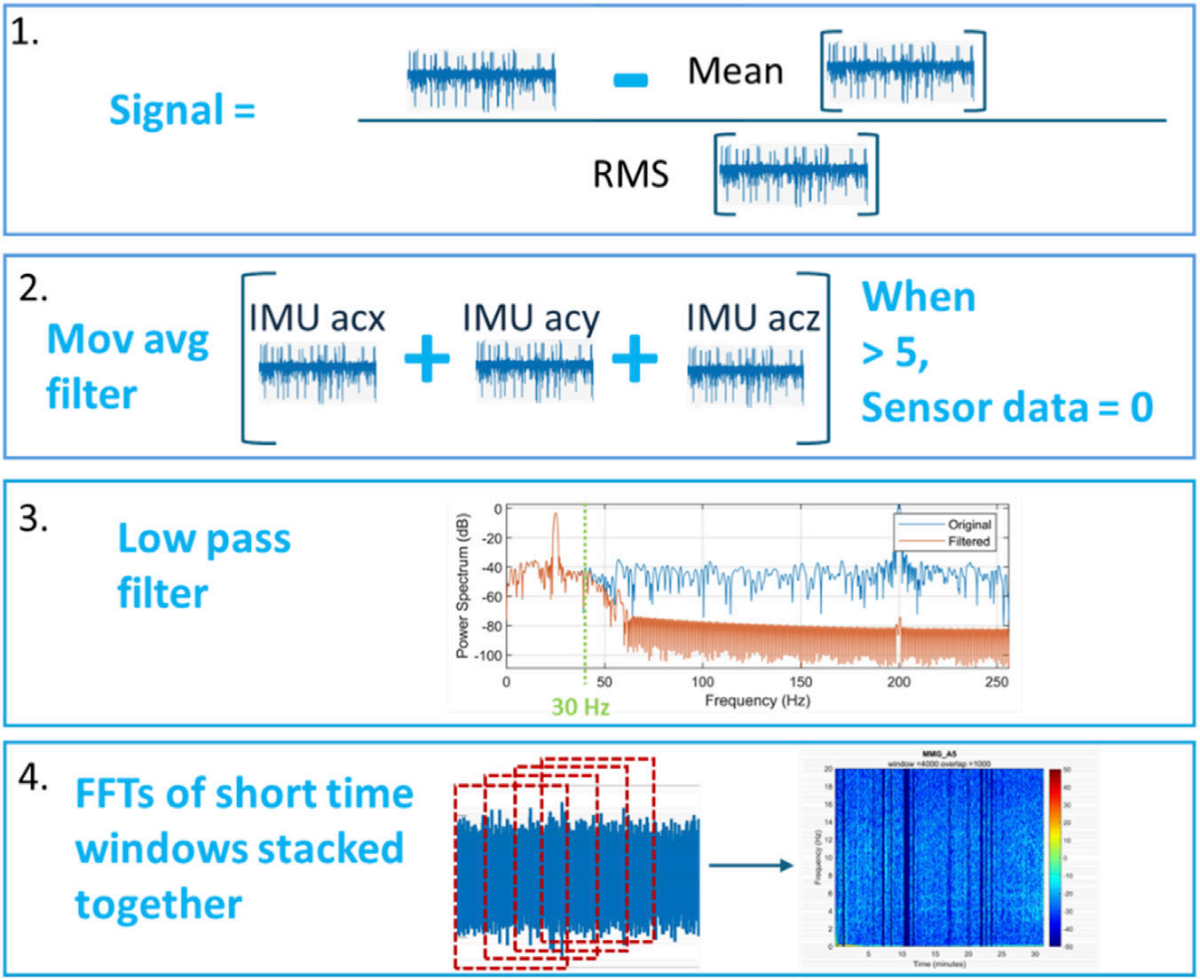
Preprocessing steps carried out before ML analysis. 1. Removing the signal offset, 2. Cancelling noise due to ultrasound probe movement, 3. Removing out of scope frequencies and 4. Generating STFTs from the cleaned signal.

Finally, it is well-documented in the literature that most of the fetal movements occurs within a frequency range of up to 30 Hz (Ghosh et al., 2024). Therefore, a low-pass filter was applied to all sensor data to eliminate frequencies above 30 Hz. MATLAB’s in-built low pass function which utilises a Finite Impulse Response (FIR) filter using Kaiser window was used for implementation with a sampling frequency of 512 Hz. The filter Bode plot is shown below in Figure 2. Figure 3 details all the data preprocessing steps.

### 3.2 ML model inputs

The pre-processed signals were subjected to Short Time Fourier Transform (STFT) analysis using three different data input methods. Given that the data signals are recorded from six different sensors, we performed individual, concatenated, and summed sensor data analysis, each of which are explained in detail in the following sections. The time window for the STFT analysis was set to 8 s, with a 2-s overlap between the windows. These parameters were chosen as they produced the best results after performing grid search hyperparameter optimisation iterating the time window from 2 to 10 s and the overlap from 1 to 4 s. This gave the STFT a frequency resolution of 0.125 Hz.

#### 3.2.1 Model input 1: individual sensor data analysis

**FIGURE 4:**
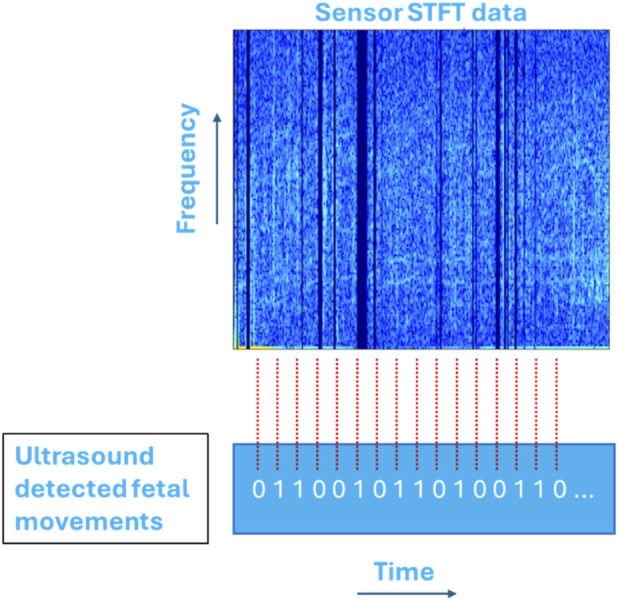
Model 1: Performing ML analysis on individual sensor data. Afterwards, all six sensor confusion matrices were averaged to evaluate their performance.

In this analysis, STFT was performed on each signal, and the resulting spectrograms were used for machine learning. The performance of each sensor was evaluated individually as in Figure 4, and the overall performance reported is the average of all six sensors. Analysing each sensor separately preserved the unique characteristics of each signal and helped identify the sensitivity of each sensor. Additionally, the reduced dimensionality of the data allowed for faster computation for each signal and decreased the likelihood of overfitting. However, averaging the final results may have diluted some critical information captured by individual sensors.

#### 3.2.2 Model input 2: concatenated sensor data analysis

**FIGURE 5:**
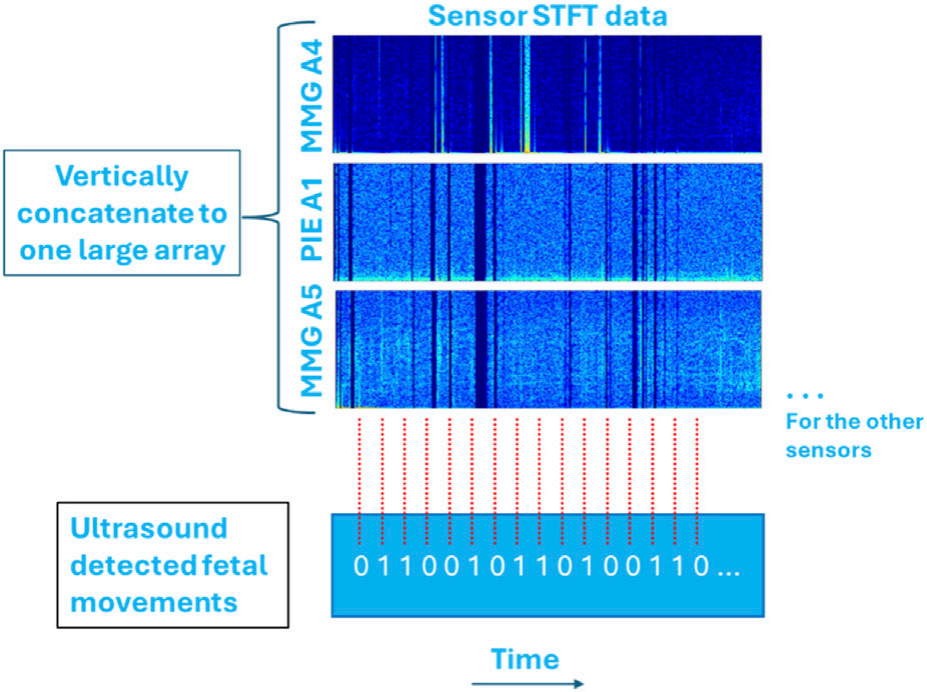
Model 2: Concatenating all STFT arrays vertically to form one large array.

This data analysis involved performing STFT on each sensor’s data to obtain separate spectrograms, which were then concatenated vertically into one large array as represented in Figure 5. The concatenated spectrogram was used as input for machine learning analysis. This method helped retain the unique features of each signal, whereby larger dimensionality of input data leads to more accurate machine learning models. Additionally, stacking the data allowed the machine learning model to learn features from each signal type separately and combine them in a meaningful way. However, higher dimensionality also increased the computational load and the risk of overfitting.

#### 3.2.3 Model input 3: summed sensor data analysis

**FIGURE 6:**
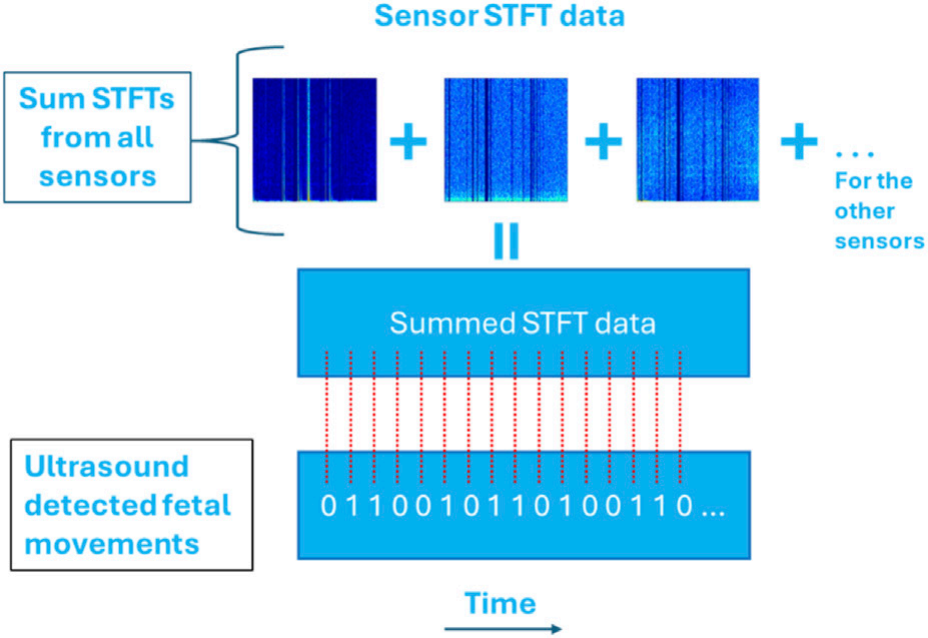
Model 3: Generating STFTs for each sensor and adding them to form a new summed STFT array. The ultrasound detected fetal movements are synched with the new STFT for ML analysis.

Summed sensor data analysis involved generating STFTs for all six sensors and summing them to form a new STFT, as demonstrated in Figure 6. The resulting STFT was then used for machine learning analysis. This combined signal contained frequency representations from all six sensors, and the model benefited from reduced dimensionality while still incorporating information from all signals. Additionally, combining the signals may have provided new insights that individual sensors might not capture. However, this method may cause certain characteristics of one signal to overshadow those of another, potentially resulting in a spectrogram that does not fully represent the original properties of any single signal.

### 3.3 Machine learning algorithms

The processed data was then correlated with the UDFM. The actions were simplified into two possibilities, movement, or non-movement. This effectively reduces the problem to a classification problem with two classes. To approach this, a few common machine learning classification techniques were considered, before finally selecting RUSboosted ensemble trees as the most effective. These different algorithms are discussed here.

**FIGURE 7:**
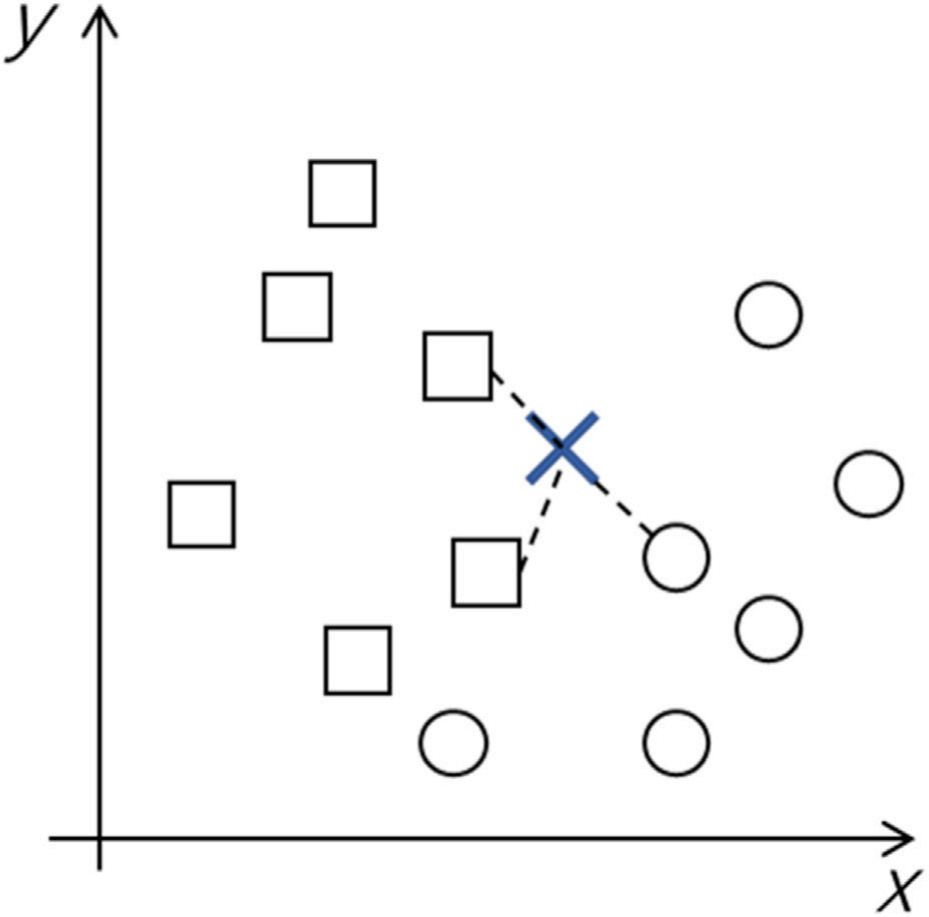
Example of KNN techniques to identify classes in a 2D plane. Here, the two classes available are squares and circles. The query point is predicted based on the three closest points.

K nearest-neighbour (KNN) is a supervised machine learning classifier. It predicts a classification query by comparing it to nearby data points within the training set. The class which most of the nearby data points belong to decides the predicted class for a query point. This works on the assumption that the points of the same class can be found near each other within the feature space. Hyperparameters which can be adjusted to improve model performance include K, the number of nearest data points used, the distance metric used to determine which points are the closest, and distance weightings. Generally, an odd number is chosen for K to avoid a tie between different classes, but the optimal value for K depends on the inherent nature of the data and the amount of training data available. The most popular distance metric is a Euclidean metric which is the true straight line distance between two points in Euclidean space. Other popular metrics include Minkowski distance, Manhattan distance and Chebyshev distance. An example of KNN to distinguish between two classes, circles and squares, within a 2D x-y plane is shown below in Figure 7. A query point ‘X’ in the 2D plane is compared against its three closest neighbours. In this case, two of its neighbours are squares, and one is a circle. As the squares have the majority, the query point is predicted to be a square.

**FIGURE 8:**
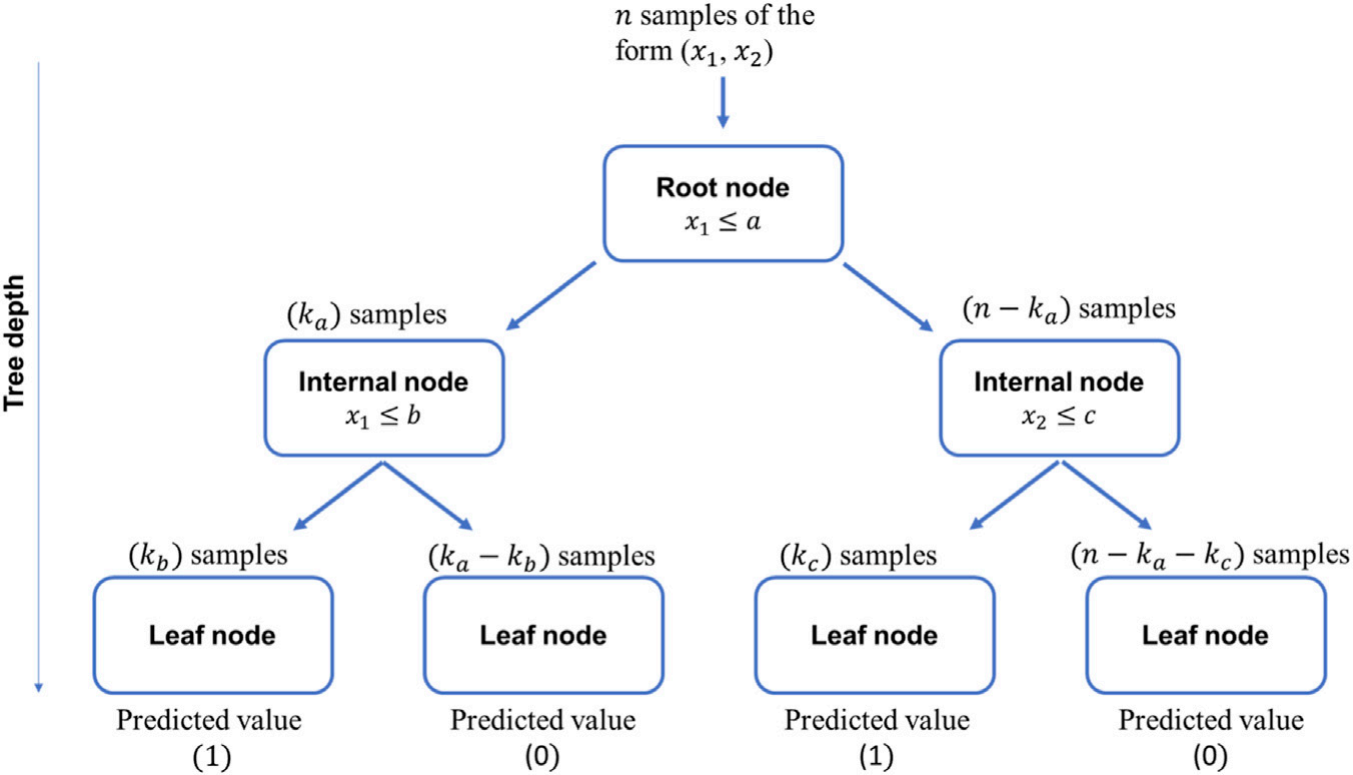
Example of a 2-layer depth decision tree to predict classes 0 and 1.

Another common method involves using decision trees, whereby data is passed sequentially through a series of nodes. The first node is known as the root node, which splits the data into two sets based on some criterion. Each split of the data will then pass through a series of internal nodes which each further split the data based on their own criterion, forming a tree-like structure until they reach a decision or leaf node. Based on the criterion at the leaf node, a prediction is made. The depth of the model is considered the number of internal node layers the data must go through before reaching a leaf node (De Ville B. Decision trees. Wiley Interdisciplinary Reviews, 2013). Figure 8 shows an example of a decision tree of depth 2, for some input data based on two variables $x_{1}$ and $x_{2}$ , for a two-class classification problem with outputs being either 1 or 0 . In this example, each node splits the dataset based on a threshold criterion such as $x_{1}\leq a$ . To train the model every possible value for a is tried, noting the data split for each possibility. A criterion is used to find the best value of a which closely splits the data into the classes desired (Rokach and Maimon, 2005). Possible criterions include the information gain criterion which uses entropy measure to measure the impurity of splits. Another criterion is the Gini index, which finds the probability of all the data points belonging to the same class after the split. The split criterion is chosen based on which value of a gives the lowest Gini index. Therefore, they are trained from the top down. The split criterion processes are repeated for all the internal nodes to find the values of b and c , leaving a trained model.

Decision trees are popular for their simplicity, making them easy to implement and quick to train. They are known for being able to handle non-linearly separable problems, and they are also easy to adapt to multi-class problems and regression problems. To make decision trees more accurate the depth of the trees can be increased.

Some models even work with many trees in parallel, where each tree makes a prediction, and a majority vote is taken from all trees to submit the final prediction. These are known as ensemble trees, and it has been shown that highly accurate classifiers can be formed by combining votes from individual classifiers in this way (Dietterich, 2000). There are many techniques for doing this, such as boosting (related to Adaboost algorithms), or bootstrap aggregation, also known as bagging (Breiman, 1996).

Ultrasound detected fetal movements created a large class imbalance in the data. For most scans fetal activity was spread and inactivity skewed the data towards non-active time stamps. It can be challenging for most classification models to deal with unbalanced data. However, there are some techniques designed to deal with data imbalances. Common techniques involve Oversampling and Undersampling which attempt to even out the imbalance, where either example are added to the minority class (Oversampling) or they are removed from the majority class (Undersampling).

These are often combined with boosting techniques to improve classification performance. Boosting involves creating an ensemble of many classifiers, which are combined by taking a majority vote. This is based on the idea that a set of many weak learners can together create a single stronger learner, as long as the weak learners are at least slightly better than random guessing. One of the most popular boosting algorithms is Adaboost (Adaptive Boosting) (Freund and Schapire, 1996). It develops an ensemble of many weighted decision trees iteratively, where each tree adds to the identified losses of the previous iteration. Starting with a pre-defined set of trees, with equal weights, where each tree is a weak learner, each iterative step involves training a tree and checking its performance. The misclassified examples are then given more weight and prioritised in the next iteration, training the next tree. Therefore, each new tree targets the errors of the previous trees, making a sequence of many weak learners. To make a prediction, results are combined from all the trees through weighted voting, where more accurate trees are given more influence.

Another boosting method, specifically for imbalanced classification problems is RUSBoost (Random Undersampling Boosting) (Seiffert et al., 2008). This introduces data sampling techniques to the traditional Adaboost algorithms, taking inspiration from SMOTEBoost (Synthetic Minority Oversampling Technique) (Chawla et al., 2002) but aiming to reduce its complexity and training time without compromising performance. This is done by randomly removing data samples from the majority class, as opposed to extrapolating minority class examples using KNN techniques. The resulting model therefore has less data to train on, improving the model processing times.

## 4 Results

All three models were trained on 38 different scans to evaluate their performance. We used 70% of the data for training and the remaining for prediction. To begin the analysis, an IMU threshold had to be decided to effectively cancel out the noise due to the probe movement. A sample dataset with a maximum number of UDFM was selected to evaluate the threshold. The model was run for threshold values of 4,5, and 6, precision and recall were selected as the performance evaluation parameters. IMU value of five yielded the best results with precision and recall of 0.29 and 0.41, respectively. A value of four reduced the recall to 0.35, whereas a value of six reduced the precision to 0.27.

Precision, Recall and F1 score were the two main parameters used to classify the performance of the model. These parameters were calculated using Equations 1–3 respectively. Precision was defined as the number of times the model predicted there was a movement picked up by sensors against all true movements identified by ultrasound. Whereas Recall was defined as the number of times the model predicted there was a movement picked up by sensors against the true sensations identified by the model. F1 score is calculated as the harmonic mean of Precision and Recall. A high performing model will have a Precision, Recall and F1 score close to 1. In this context, a high Precision model can identify all UDFM and periods of no UDFM correctly. A high Recall value model will be a highly sensitive model that will not predict a fetal movement if there is no movement and *vice versa*. A high F1 score implies both a high Precision and Recall value, and that these values are close to each other.

$$Precision=\frac{TP}{TP+FP}$$ (1)

$$Recall=\frac{TP}{TP+FN}$$ (2)

$$F1\;score=\frac{2\times Precision\times Recall}{Precision+Recall}$$ (3)

Code for machine learning models was written in Matlab. To compare the different classification methods against each other, the individual sensor SFTFT data from one scan was used to train a model with each method. The precision and recall of each model were found and then plotted against each other to find the best model algorithm for this problem.

**FIGURE 9:**
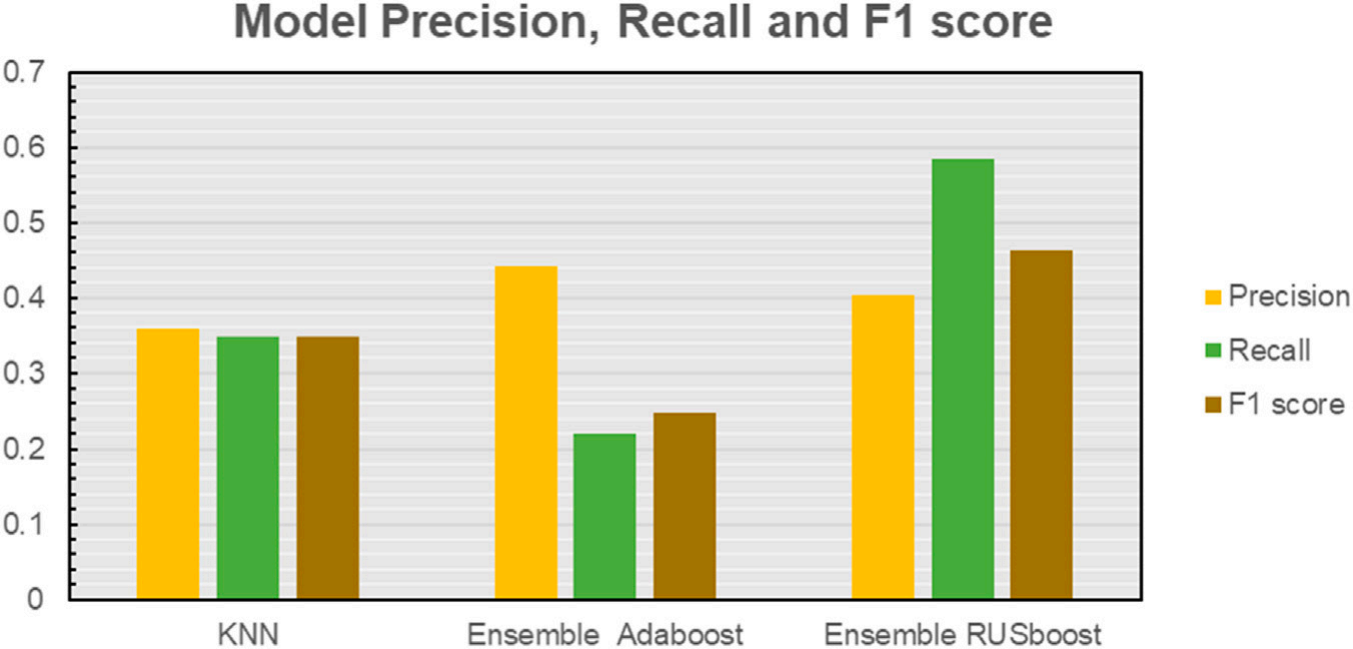
Comparison of different ensemble methods.

Figure 9, Table 1 shows the results of the machine learning methods discussed here, it was found that ensemble trees with RUSboosting performed the best for our dataset, showing the highest average of Precision and Recall. This model was then continued for all the data input arrays described in Section 3.2.

**TABLE 1:** Performance data metrics for the classification models.

Model/Metric	Precision	Recall	F1 score
KNN	0.359	0.348	0.348
AdaBoost	0.441	0.220	0.249
RUSBoost	0.404	0.584	0.464

**FIGURE 10:**
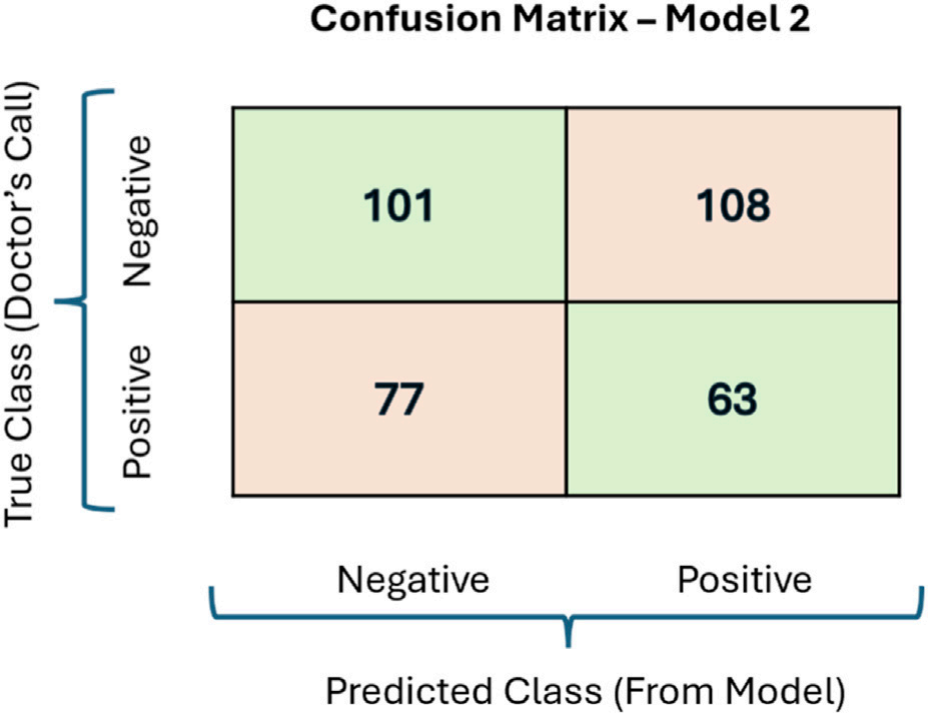
A sample confusion matrix generated by model 2. Precision and recall are calculated from the values of these confusion matrices to evaluate the performance of a model.

To evaluate the performance of the three model inputs, confusion matrices were used. For model input 1, the matrices were generated for individual sensors after which True Positive (TP), True Negative (TN), False Positive (FP) and False Negative (FN) values were averaged respectively to get the performance parameters for one subject, later all the individual subject’s confusion matrices were averaged to get the model performance. Model input two involved concatenating all six STFT sensor data, a single confusion matrix was generated for the concatenated data, and then all the subject confusion matrices were averaged to arrive at the final result. For Model input 3, data from all six sensors were used to generate individual STFTs, which were then combined to form a single STFT of the same dimensions. This composite STFT was used in the machine learning analysis to generate the confusion matrix. Further, similar to model input 2, all the participant’s confusion matrices were averaged to evaluate the performance of the model. Figure 10 shows a confusion matrix generated from a subject using model 2 as an example.

**FIGURE 11:**
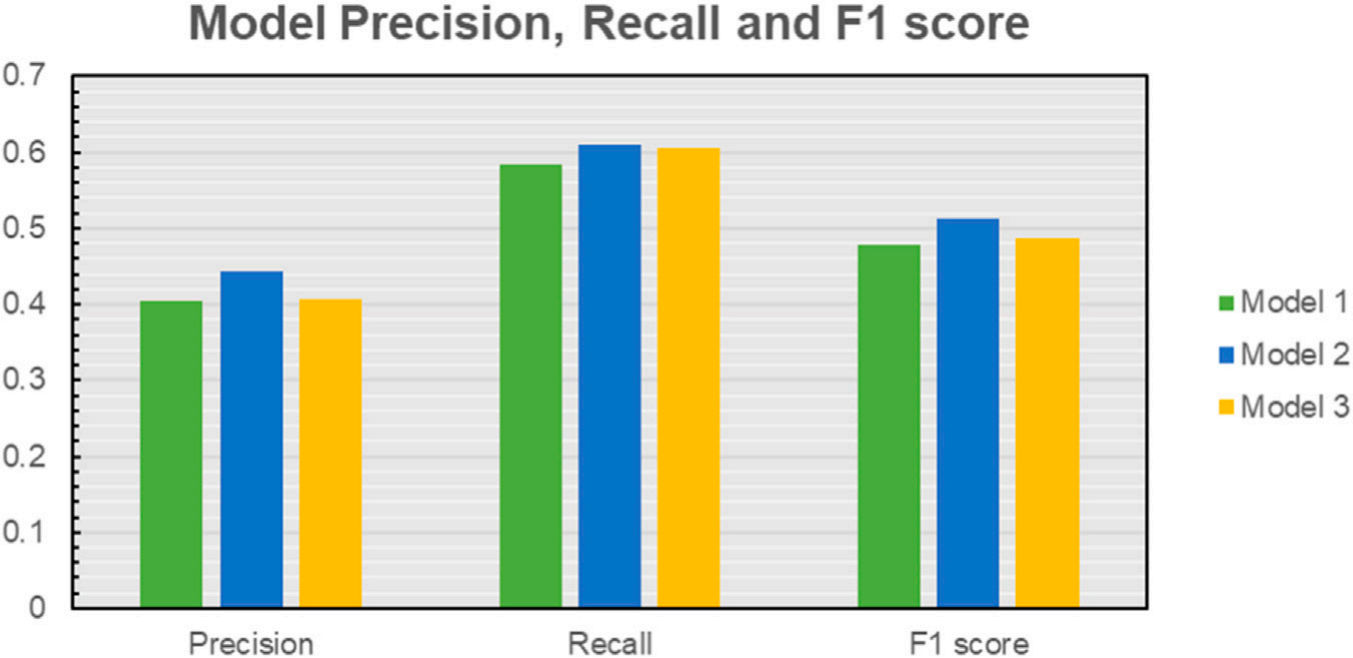
Precision, Recall and F1 score for all three models. It is observed that all models have ML performance parameters in the same range with Model two outperforming the other two by a small margin.

After the analysis, it was observed that all three models had similar values of Precision, Recall and F1 score in the range of 0.41–0.44, 0.58–0.61, and 0.48–0.51 respectively, as shown in Figure 11 and Table 2. Model 2, which concatenated the STFTs of all six sensors, had the best performance out of the three with a Precision, Recall and F1 score of 0.44, 0.61, and 0.51. Model one evaluated the performance of each sensor and then averaged them to predict the results. It is possible that in this process of averaging the results of sensors, the model might have diluted the effect of the main sensor that detected the response. For Model 3, which involved adding the STFTs of each sensor, it is possible that in the process of adding all the spectrograms, the final STFT might have missed the characteristics of individual sensors. Model 2, which concatenated and stacked the arrays, had all the information without any alteration and averaging, which might have led the model to outperform models one and three by a small margin.

**TABLE 2:** Performance data metrics for the classification models.

Model number/metric	Precision	Recall	F1 score
Model 1	0.404	0.584	0.478
Model 2	0.443	0.611	0.514
Model 3	0.406	0.606	0.487

## 5 Discussion

**FIGURE 12:**
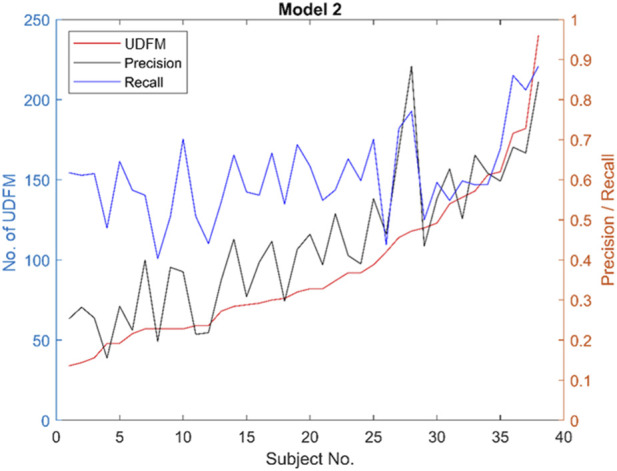
A direct correlation between the number of UDFM and ML performance parameters. The results suggest that the higher the number of data points higher the ML performance.

The data collection for this analysis was done over a period of 30 min, and the ultrasound detected fetal movements were used for training the ML algorithms. In total, from the 38 scans (through which the ML was trained) the maximum number of ultrasound detected fetal movements that we could obtain was 240. Each of these activities lasted about 1s, which meant out of the 1800s of data collection, only 13% of the time the ML algorithm had active data to train on. The heavy skewness towards the non-active data might have caused the performance of the ML algorithms to drop. This is substantiated from the individual analysis carried out on participants to evaluate precision and recall. Figure 12 demonstrates the relationship when the individual patient scans are sorted based on the number of movements detected by ultrasound and compared to precision and recall. This shows that precision and recall increase with a greater number of UDFM, until the UDFM threshold remains in the same range; however, after the threshold, recall takes a sharp increase with UDFM. This trend of increasing performance parameters for ML model suggests that wearable devices that can be used for an extended period of time will potentially produce better results.

Future studies could therefore focus on collecting a larger dataset. A higher proportion of positive fetal movement data points would make techniques such as undersampling and oversampling the data imbalance more effective in improving the model performance. Other strategies to improve model performance in future work could involve more complex machine learning algorithms, such as convolutional neural networks. Modelling techniques using neural networks can combine sensor data in different ways than summing or concatenating data arrays as done here, which may lead to more powerful model predictions.

Other considerations for future work include exploring the impact of sensor placement and sensor type. In this work sensors have been distributed across the whole abdomen area, and data has been collected from all sensors simultaneously. A mixture of acoustic and piezoelectric sensors has been used, and data from all sensors has been collected as an input into the machine learning models. Future work could analyse the contribution of each individual sensor to the model performance, allowing us to remove sensor positions/types which do not greatly improve the model. Simplifying the sensor setup would bring us closer to an inbuilt sensor monitoring belt design, with optimised sensor placement ready to be used in real-world, home-use cases.

The final FM ensemble RUSboost prediction model had a precision and recall of 0.44 and 0.61. Comparing to similar models in literature, KAPPA coefficient metrics are often used. KAPPA coefficients are within the range of −1 to 1, with −1 indicating perfect disagreement, 0 indicating no correlation and one indicating perfect agreement (Ryo et al., 2012). recorded a KAPPA coefficient of 0.57 and 0.71 at 20–29 weeks and 30–39 weeks respectively (Nishihara et al., 2008). also recorded a KAPPA coefficient of 0.75. These statistics are close, although not directly comparable as these studies use different sensors for recording the data and concentrate on specific fetal activities to predict the results. However, with the future work strategies discussed it is expected the performance of the proposed model will further improve.

**FIGURE 13:**
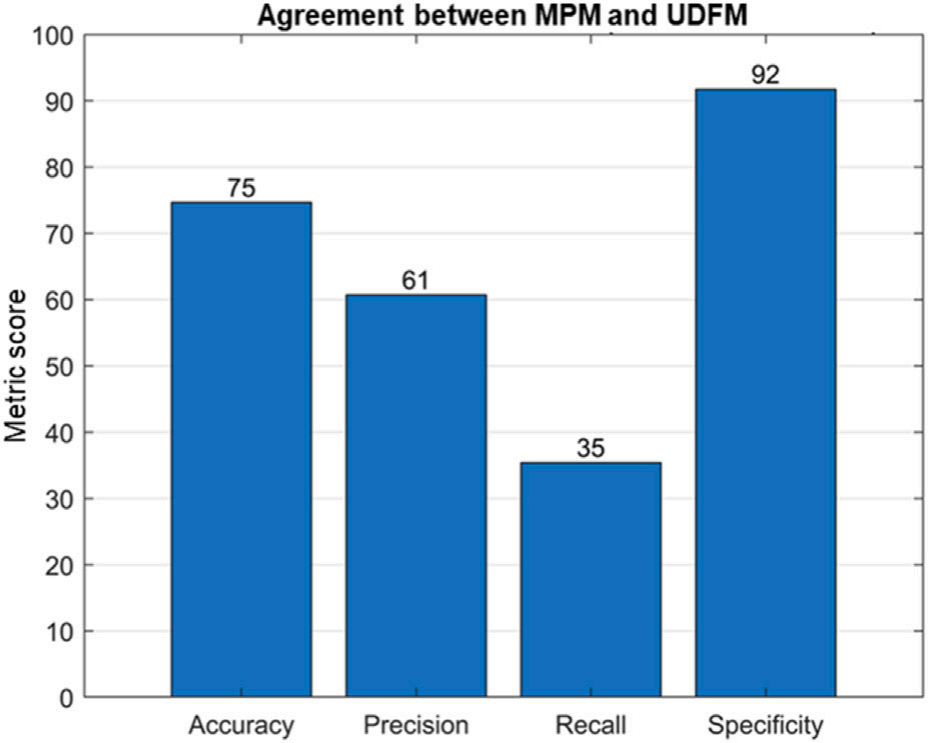
Agreement between MPM and UDFM data.

The relationship between UDFM and Maternally Perceived Movements (MPM) was also investigated. Figure 13 shows the results of this analysis. In this context, accuracy is defined as the number of times UDFM and maternal perception agree. Precision shows that 61% of movements detected by ultrasound were also perceived by the pregnant participant. However, a low recall value indicates that many movements detected by ultrasound are not maternally perceived. This result is in accordance with previous research findings (Hijazi and East, 2009; Cito et al., 2005) that showed not all fetal movements are perceived by pregnant women. Finally, a high specificity shows that ultrasound and maternal perception are similarly accurate in detecting no movement. The reason for high specificity may also be due to the high skewness of training data towards non-active movements.

## 6 Conclusion

In this work, we performed ML analysis on 38 number of scans recorded at 32–34 and 36–38 weeks of gestational age. Different ML techniques and data preparation methods were employed to evaluate the best performing model. From the analysis performed following conclusions can be drawn.

1. Among KNN, decision trees, ensemble AdaBoost and ensemble RusBoost, the ensemble RusBoost ML algorithm produced the best results and was used to evaluate the performance of three different data models.
2. The three data preparation methods: Individual, Concatenated and Summed sensor data produced results in a similar range, with concatenated data outperforming the others by a narrow margin, which can be attributed to training on real data without any modification.
3. Model performance parameters, precision and recall had a direct correlation to the active data. It was observed that both precision and recall increased with the number of active movements in the test data.
4. It was noted that pregnant people do not perceive all the fetal movements detected on ultrasound. UDFM identified a significant number of movements that were not perceived maternally.

The present study shows promising results that fetal monitoring can be performed with the help of Piezoelectric and our custom-packaged acoustic sensors. The performance of the ML model can be improved by increasing the number of participants analysed, thereby recording more active movements of the fetus and increasing the power of the study. It is recognised this sample size is small however, this represents a novel, exploratory study to see the feasibility of applying these techniques on a larger scale. Moreover, understanding that many movements are missed by maternal perception compared to objective ultrasound detection emphasises the importance of external wearable devices during pregnancy to monitor fetal health.

## Data Availability

The raw data supporting the conclusions of this article will be made available by the authors, without undue reservation.
