# Supplementary material for: A machine learning model for assessing fetal health during pregnancy
Source: Front Bioeng Biotechnol. 2025 Dec 17;13:1691064. doi: 10.3389/fbioe.2025.1691064 (PMC12754730; doi:10.3389/fbioe.2025.1691064)
Supplement: Supplementary file 1 [file DataSheet1.docx]

## Appendix 1

The health data of the participants of this study is reported in Figure A1 and a real time testing image is shown in Figure A2.

*Fig A1. Health data of participants enrolled for this study.*

*
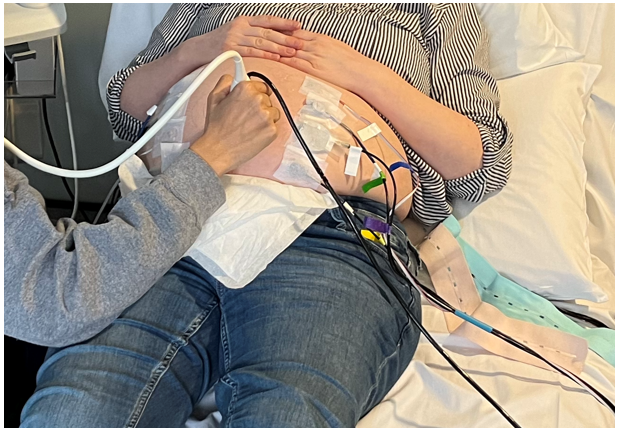
*

*Fig A2. Sensors attached to a participant’s abdomen during data collection.*
